# State-of-the-art treatment of postpartum bipolar disorder

**DOI:** 10.1097/YCO.0000000000001049

**Published:** 2025-10-15

**Authors:** Tamás Kurimay, Anett Pelikán, Vera Tory

**Affiliations:** a Buda Family Centred Mental Health Centre, Department of Psychiatry and Psychiatric Rehabilitation Teaching Department of Semmelweis University, Together, Baby-Mother-Father Unit, North-Buda Saint John Central Hospital; b Buda Family Centred Mental Health Centre, Department of Psychiatry and Psychiatric Rehabilitation Teaching Department of Semmelweis University, Together, Baby-Mother-Father Unit; c Head of Pediatric and Neonatology Department, North-Buda Saint John Central Hospital, Budapest, Hungary

**Keywords:** lived experience, mother–infant bonding, pharmacotherapy, postpartum bipolar disorder, relapse prevention

## Abstract

**Purpose of review:**

To summarize recent updates in the treatment of postpartum bipolar disorder (PBD).

PBD requires timely and comprehensive management, as childbirth is a period of elevated relapse risk in women with preexisting illness and may also mark first-onset presentations. Episodes can manifest as depression, mixed states, mania, or psychosis, with severe consequences for maternal safety, infant well being, and early bonding. Sleep loss around labor and postpartum further increases vulnerability.

**Recent findings:**

PBD demands a multifaceted therapeutic approach, with pharmacotherapy as the cornerstone. Lithium, lamotrigine, and selected second-generation antipsychotics remain key options, guided by efficacy, lactation safety, and individualized risk–benefit assessment. Structured relapse-prevention planning, sleep protection, and support for mother–infant bonding are crucial nonpharmacological elements. Early, targeted intervention reduces relapse and suicide risk while supporting maternal functioning and family stability.

**Summary:**

Beyond optimized pharmacological care, recent research highlights a treatment continuum spanning pregnancy and postpartum. Multidisciplinary collaboration across psychiatry, obstetrics, and neonatal care is crucial to ensure maternal safety, optimize infant outcomes, and support families. Integrating lived experience and patient collaboration enhances relevance. A life-course perspective across reproduction, combining biological and psychosocial insights, signals a shift toward holistic, personalized, precision-based strategies in managing PBD.

## INTRODUCTION

Perinatal mental health (PMH) has become a public-health priority: morbidity and mortality data underscore its burden, and many countries have implemented specialized services [1].

Bipolar disorder is a lifelong, clinically and genetically heterogeneous illness [2], with variable severity, multiple subtypes, and region-specific prevalence [3]. Lifetime prevalence is 0.6% for BD-I and 0.4% for BD-II, rising to 1–2.4% with the broader spectrum [3,4]. Age of onset shows a trimodal distribution: early-onset (45%) peaking at 17 years, mid-onset (35%) at 26 years, and late-onset (20%) at 42 years [5], overlapping with reproductive and parenting years.

Management of perinatal mental disorders (PMDs) should be viewed from preconception through pregnancy and childbirth to the postpartum period and the parenting years. The perinatal period carries increased vulnerability, with risk of both relapse and first-onset PMD. Psychiatric hospitalization in the first postpartum month is 22 times higher than prepregnancy, mainly for mood and psychotic disorders [1,6].

The course of bipolar disorder has been linked to hormonal changes across the female reproductive cycle [7], including the perinatal period, where relapse risk of bipolar disorder reaches 39–70% with a prior diagnose, while bipolar spectrum mood episodes occur in 16–24.5% of women without psychiatric history [8].

Severe bipolar disorder episodes in the perinatal period often require hospitalization and carry risks of self-harm, infant harm, and impaired parenting. Pharmacological management must be individualized: considering prior response, timing, fetal and neonatal risks, and lactation safety [9^▪▪^].

Arbitrary treatment discontinuation often compromises adherence and heightens relapse risk [10^▪▪^]. Care is influenced by family and social context, and optimal outcomes depend on multidisciplinary collaboration across healthcare, social services, and patients [11].

Definitions of postpartum episode vary: DSM-5 sets 4 weeks [12], ICD-11 6 weeks [13], while recent literature often extends to 12 months. 

**Box 1:**
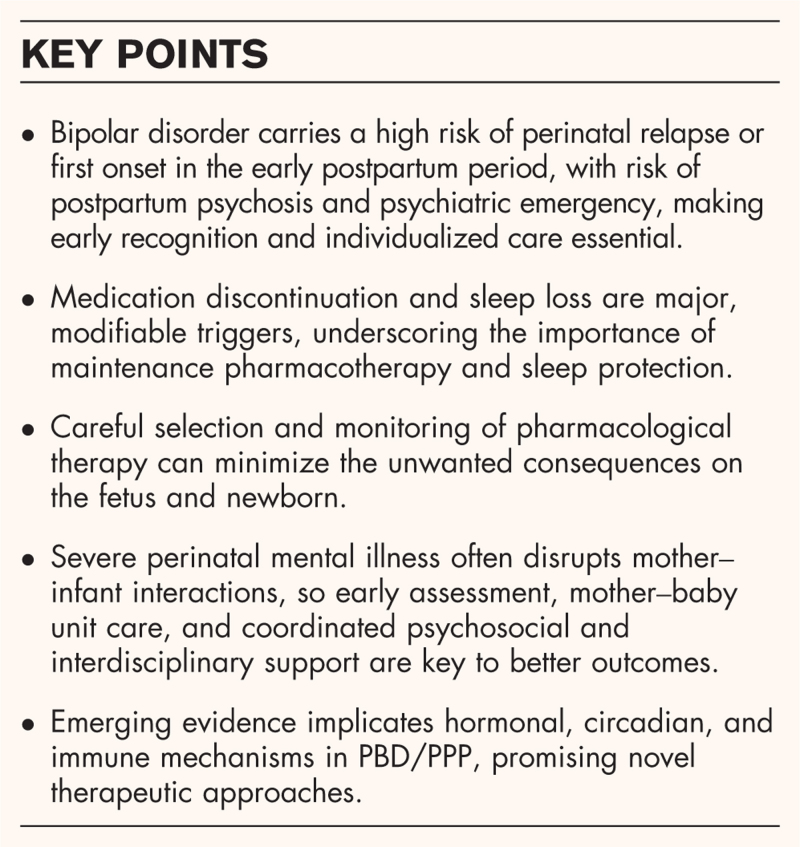
no caption available

## MATERIALS AND METHODS

We searched PubMed, PsycINFO, Cochrane, Google Scholar, and Web of Science (January 1, 2024–submission) for perinatal/postpartum bipolar disorder, focusing on treatment and management; after de-duplication, three reviewers screened full texts and included peer-reviewed studies, guidelines, and consensus statements.

## PURPOSE OF REVIEW

This review synthesizes recent evidence, emerging trends, and key challenges in PBD within the perinatal continuum.

## EPIDEMIOLOGY OF POSTPARTUM BIPOLAR DISODER

A recent meta-analysis found that postpartum mood episodes occurred in 29–49% of women with BD-I, with 27–50% presenting with manic or mixed episodes. Maintenance pharmacotherapy reduced relapse risk from 58.1% (untreated) to 25.9% (treated) [14].

Further data confirm high perinatal recurrence rates: 57% for BD-I or schizoaffective bipolar disorder and 62% for BD-II or other bipolar disorder, with around 40% substantial relapse during pregnancy. Postpartum episodes of mania/psychosis in more than 90% of women began within 4 weeks of delivery. Psychosis was six times higher in BD-I/SA-BD [15].

Peripartum sleep deprivation elevates early-onset postpartum psychosis (PPP) risk: losing one full night is reported to increase odds over 5-fold. 92% of PPP episodes occurred within two weeks, and all episodes within three weeks. Onset was more variable without sleep loss [16].

Fathers with bipolar disorder are also at risk for perinatal mood episodes with distinct timing of onset compared to mothers, most often during pregnancy (41.9%), whereas mothers within the first week (41.3%) [17].

The presence of bipolar disorder is one of the main risk factors of PPP. Personal and family history of psychotic disorders, sleep deprivation, and discontinuation of medication pose further risk [18^▪▪^].

In the MGHP3 study of PPP, onset occurred a median of 10 days postpartum, episodes lasted 1 day–1 month in 60.9%. BD-I was presented in 71.8%. Overall, 93% received medication, 74% were hospitalized, underscoring severity, acute onset, and need for intensive management [19].

Postpartum rage attacks are often associated with bipolar disorder, OCD, or depression. This form of dysfunctional anger requires screening for first-onset bipolar disorder and identification of potential triggers such as antidepressants or sleep loss [20]. A recent study found that first-onset SMI occurred within 12 weeks postpartum, with 25% of acute transient psychotic disorders converting to bipolar disorder [21].

## PATHOPHYSIOLGY OF POSTPARTUM PSYCHOSIS AND POSTPARTUM BIPOLAR DISORDER

The uniqueness of postpartum mental disorders remains debated. Early-onset postpartum depression (PPD) and PPP may be bipolar disorder linked subtypes, often characterized by mixed symptoms. PBD may represent a childbirth-triggered, pathoplastic variant of bipolar disorder with distinct clinical profiles and treatment needs, supporting the importance of tailored management [22].

Reproductive hormonal fluctuations have been implicated in shaping the course of BD. In a recent study of 567 postmenopausal women with bipolar disorder (BD-I 72.1%, BD-II 22.4%), premenstrual syndrome [odds ratio (OR) 6.19] and PPD (OR 2.64) predicted perimenopausal depression, while premenstrual dysphoric disorder predicted any mood episode (OR 3.01) and depression (OR 3.03), suggesting a biologically sensitive subgroup of bipolar disorder vulnerable across reproductive transitions [23].

Psychosocial factors play an important role in PMH. In PPP, biological factors are likely to be central. Genetic data distinguish early-onset and late-onset PPD, with early-onset overlapping major depressive disorder (MDD) and bipolar disorder, shows enrichment of genes expressed in reproductive tissues, suggesting hormonal mechanisms [24]. PPP shows high heritability, and overlapping with bipolar disorder, schizophrenia, and autoimmune disease [25]. Inflammation-related genetics are central in MDD-PPD and circadian/sleep-related pathways predominate in BD-PPD [26]. PPP is conceptualized as an interaction among genetic vulnerability, hormonal changes, and environmental factors, with bipolar disorder and circadian disruption emerging as key contributors [27^▪^].

## RISK OF POSTPARTUM BIPOLAR DISORDER

Recent data confirm increased risk of gestational diabetes (OR 1.46), hypertension (OR 1.19), antepartum hemorrhage (OR 2.02), preeclampsia/eclampsia (OR 1.20), cesarean section (OR 1.35), and postpartum hemorrhage (OR 1.39) in women with BD. Neonatal risks included very prematurity (OR 1.84), infant death (OR 1.77), low birth weight (OR 1.54), preterm birth OR 1.49), small for gestational age (OR 1.28), and congenital malformations (OR 1.29) [28].

Maintenance treatment in bipolar disorder plays a critical role in preventing relapse and reducing perinatal risk, with growing evidence of benefits over nontreatment. Cohort data of 198 infants indicate that lamotrigine, lurasidone (and SGAs) reduce risks of prematurity and low birth weight, compared to untreated disorder [29].

Women with bipolar disorder face elevated obstetric and neonatal risks, necessitating multidisciplinary perinatal care and shared decision-making with obstetrics and neonatology. Given the risk of neonatal adaptation syndrome, neonatal assessment and observation are essential, requiring close collaboration with neonatologists.

## MOTHER INFANT INTERACTIONS IN BIPOLAR DISORDER

In women at risk for PPP (bipolar disorder, schizoaffective disorder, or prior PPP), predictors of mother-infant interaction (MII) quality were assessed up to 12 months postpartum. In women at risk for PPP, impaired maternal fear recognition predicted poorer MII [30].

PPD can negatively impact child's development through MII. Assessment of 40 six-month-old infants of mothers with severe PPD found global developmental delay, poorer sociability and postural development. Infants of mothers with bipolar disorder showed significantly poorer postural development [31].

Mother–infant interactions are often impaired in mothers with SMI. A recent review found impairments in bipolar disorder, more pronounced in PPP [32].

The authors emphasize early evaluation of MII and infant development, underscoring the need for access to MBUs, facilitated contact that supports attachment, early interventions and targeted support to improve bonding and developmental outcomes.

## MANAGEMENT OF POSTPARTUM BIPOLAR DISORDER

Bergink *et al.* [10^▪▪^] recommend a perinatal relapse prevention plan, covering treatment options, preferred mode of delivery and infant feeding, postpartum relapse prevention, ensuring adequate sleep, and recognizing early signs of relapse.

Bipolar disorder is a main risk factor of PPP [18^▪▪^], characterized as a psychiatric emergency requires urgent pharmacological treatment and inpatient-care, given symptoms unpredictability. MBU's are preferred, while supervised access to the infant may be arranged in general psychiatric settings. Management should be individualized, with immediate goals of symptom control and risk reduction, evaluation of suicide and infanticide risk, and long-term aims of strengthening maternal role and mother–infant bonding [33].

An integrated model of obstetric, perinatal, and psychiatric care enables routine screening and interdisciplinary management of PMDs, prioritizing high-risk cases such as bipolar disorder and PPP [34].

Maintenance treatment during pregnancy has to be considered even in euthymic patients [35^▪▪^]. In bipolar disorder without maintenance treatment, postpartum prophylaxis should be considered to prevent relapse [10^▪▪^]. It remains unclear whether initiating prophylaxis after childbirth is sufficient, or starting 2–4 weeks before delivery is optimal [33].

## PHARMACOLOGICAL TREATMENT

### Valproate, carbamazepine

#### Pregnancy and peripartum

VPA and CBZ should be avoided in women of childbearing potential due to high teratogenic and neurodevelopment risks [9^▪▪^,^10^^▪▪^,^35^^▪▪^]. UK guidance also restricts VPA use in men [36], while these restrictions raise concerns on patient rights and treatment adherence [37^▪^]. Alternatives of VPA include quetiapine, olanzapine, aripiprazole; and haloperidol in acute phases, and lamotrigine for depression prophylaxis. Lithium remains an option in acute/maintenance treatment, with careful monitoring [38^▪^] (Table 1).

**Table 1:** Pharmacotherapy in perinatal and postpartum bipolar disorder overview (original table based on the literature review)

Class	Agents	Potential benefits	Main risks and concerns	Clinical considerations
Mood stabilizers	Lithium [9^▪▪^,^10^^▪▪^,^35^^▪▪^,^40^^▪▪^,^43^,^44^]	Gold standard; effective in acute/maintenance treatment and suicide prevention	Cardiac/other malformations, preterm birth, LGA; neonatal adversity (irritability, somnolence, altered muscle tone, feeding difficulties, poor weight gain, renal, thyroid, cardiac impairment) [9^▪▪^,^10^^▪▪^,^35^^▪▪^,^40^^▪▪^,^43^]; Maternal, fetal/neonatal, breastfed infants’ toxicity [40^▪▪^] Possible dose-dependent risk of teratogenicity and neonatal adverse outcomes [9^▪▪^,^44^]	First-line use of agents with less teratogenic, neonatal and infant risk [35^▪▪^] Monitor maternal serum level, adjust dose as needed [9^▪▪^,^10^^▪▪^,^40^^▪▪^,^43^] Monitor fetal development [10^▪▪^] Monitor maternal preeclampsia, renal impairment, gestational hypertension [40^▪▪^] Consider peripartum dose adjustment to avoid toxicity and maintain relapse prevention [40^▪▪^] Consider postpartum initiation to prevent relapse [10^▪▪^] Consider risk of breastfeeding, monitor breastfed infants [9^▪▪^,^35^^▪▪^,^40^^▪▪^]
	Lamotrigine [9^▪▪^,^10^^▪▪^,^29^,^35^^▪▪^,^39^^▪▪^,^40^^▪▪^,^41^]	Relatively well tolerated in pregnancy/lactation in treatment of bipolar depression	Low perinatal risk [9^▪▪^,^10^^▪▪^,^40^^▪▪^] Elevated clearance in pregnancy [9^▪▪^,^10^^▪▪^,^39^^▪▪^,^40^^▪▪^] Possible dose dependent risk in pregnancy/breastfed infants [41]	Monitor maternal serum level, adjust dose as needed [9^▪▪^,^10^^▪▪^,^39^^▪▪^,^40^^▪▪^] Consider peripartum dose adjustment to avoid toxicity and maintain relapse prevention [9^▪▪^,^10^^▪▪^,^39^^▪▪^,^40^^▪▪^] Consider risk of breastfeeding, monitor breastfed infants [35^▪▪^,^41^]
	Carbamazepine [35^▪▪^,^40^^▪▪^]	Possible postpartum use	Risk of teratogenicity [40^▪▪^]	Generally discouraged in pregnancy; Breastfeeding is controversial [35^▪▪^,^40^^▪▪^]
	Valproate [9^▪▪^,^10^^▪▪^,^35^^▪▪^,^40^^▪▪^]	Possible postpartum use	High risk of teratogenicity, three-fold risk of malformations; neonatal risk, four to five-fold risk of neurodevelopmental disorders [9^▪▪^,^10^^▪▪^,^35^^▪▪^,^40^^▪▪^]	Avoid in women with childbearing potential and during pregnancy [10^▪▪^,^40^^▪▪^] Breastfeeding is controversial [35^▪▪^,^40^^▪▪^]
Antipsychotics	Olanzapine, Quetiapine [10^▪▪^,^35^^▪▪^,^39^^▪▪^,^40^^▪▪^,^46^]	Effective in acute/maintenance treatment; Safe in pregnancy/lactation	Maternal metabolic risks, obesity, GDM; risk of LGA [10^▪▪^,^40^^▪▪^] Risk of neonatal adaptation syndrome (with APs in general) [40^▪▪^]	Monitor maternal metabolic effects, fetal development and neonatal adaptation [40^▪▪^] First-line agents in breastfeeding, monitor breastfed infants [35^▪▪^,^39^^▪▪^,^46^]
	Lurasidone [10^▪▪^,^29^]	Effective in bipolar depression	Possible perinatal risk reduction (prematurity, LBW) compared to no treatment [29] Limited perinatal safety data [10^▪▪^]	Consider perinatal use with fetal, neonatal monitoring [29]
	Aripiprazole [40^▪▪^,47^▪^,^48^]	Effective in acute/maintenance treatment	Impaired lactation through prolactin lowering effect [40^▪▪^,47^▪^,^48^]	Fetal, neonatal monitoring Consider risk of impaired lactation, involve lactation consultant, monitor breastfed infants [47^▪^,^48^]
	Risperidone, Haloperidol [40^▪▪^]	Effective in acute/severe episodes	Limited perinatal safety data Risk of adverse effects in breastfed infants [40^▪▪^]	Fetal, neonatal monitoring Consider breastfeeding with caution, monitor breastfed infants [40^▪▪^]
	LAI-APs [10^▪▪^,^49^]	Improve adherence	Very limited perinatal safety data [10^▪▪^,^49^]	Consider perinatal continuation in cases of poor adherence and high risk of relapse [49] Fetal, neonatal monitoring
Antidepressants	SSRI/SNRI/other [14,40^▪▪^]	Potential use in BD-II depression; Perinatal safety	Risk of neonatal adaptation syndrome, low risk of persistent pulmonary hypertension [40^▪▪^] Risk of manic switch [14,40^▪▪^]	Avoid monotherapy, combine with mood stabilizer [14,40^▪▪^] Monitor for efficacy, and symptoms of manic/mixed states Fetal, neonatal monitoring
Benzodiazepines	Lorazepam, Loxepine, Clonazepam [10^▪▪^,^40^^▪▪^,^51^]	Rapid effect (anxiety, sleep disorders, agitation)	Low risk of adversities if used appropriate, risk of high doses in third trimester [10^▪▪^,^40^^▪▪^,^51^]	Consider perinatal use, if indicated, use with fetal, neonatal monitoring [40^▪▪^,^51^] Use appropriate dose for limited period [10^▪▪^,^40^^▪▪^,^51^]

APs, antipsychotics; GDM, gestational diabetes mellitus; LBW, low birth weight; LGA, large for gestational age; SNRI, serotonin-norepinephrine reuptake inhibitor; SSRI, selective serotonin reuptake inhibitor.

#### Breastfeeding

While some evidence suggests that VPA or CBZ monotherapy initiated postpartum may be compatible with breastfeeding without adverse effects on infant growth or development [39^▪▪^], most guidelines advise against VPA use for postpartum bipolar disorder [10^▪▪^,^35^^▪▪^,^40^^▪▪^].

### Lamotrigine

#### Pregnancy and peripartum

LTG is a well tolerated option in bipolar depression, with no demonstrated risk of malformations, obstetric/neonatal complications, or long-term neurodevelopmental effects [9^▪▪^,^10^^▪▪^,^40^^▪▪^]. Slow titration makes it less suitable for acute episodes. Increased clearance during pregnancy may necessitate two or three-fold higher doses, guided by serum levels. Postpartum, dose reduction and tapering to prepregnancy doses within the first weeks lowers risk of toxicity [9^▪▪^,^10^^▪▪^,^39^^▪▪^,^40^^▪▪^].

#### Breastfeeding

Low-moderate LTG doses are generally well tolerated in breastfed infants [39^▪▪^]. In 47 mother–infant pairs infant levels correlated with maternal dose, peaking in the first two weeks, while postpartum maternal doses decreased after delivery. Mild adverse effects occurred in 17%, with one case at 350 mg/day required weaning. No adversities were observed at doses 150 mg/day or less, suggesting a potential safety threshold [41].

### Lithium

Lithium remains the gold standard for bipolar disorder, yet its use declined in Europe [42].

#### Pregnancy and peripartum

A recent meta-analysis confirms lithium is linked to modestly increased risks of cardiac and other malformations, preterm birth, and large-for-gestational-age infants [43]. Risks appear dose-dependent – lower than with VPA or CBZ; and neonatal complications (e.g., hypoglycemia, transient thyroid/renal abnormalities) are slightly increased [9^▪▪^,^10^^▪▪^,^40^^▪▪^]. Discontinuation or dose reduction may be considered in prepregnancy planning for euthymic women with low risk of relapse. After pregnancy is established, discontinuation or medication switch risks relapse, while first-trimester dose reduction remains considerable [10^▪▪^], and continuation of lithium with close fetal monitoring and serum level assessments may help mitigate associated risks [40^▪▪^,^43^]. In 66 women with BD-I, who continued lithium into late pregnancy under a structured protocol of peripartum suspension and restart, cord:maternal ratio (1,1) showed complete placental transfer, and postpartum levels changed about 0.20 mEq/l. Early relapse occurred in 6%, while mild neonatal complications in 56%, mainly hypotonia, associated with higher neonatal levels (>0.75 mEq/l) [44].

Serum level monitoring is required given clearance-related declines and potential toxicity. Discontinuation may be indicated in preeclampsia or renal impairment, with close monitoring for gestational hypertension [40^▪▪^].

Withholding lithium for 24–48 h before delivery or implementing routine dose reduction might be controversial, as these adjustments increase relapse risk [9^▪▪^,^10^^▪▪^,^39^^▪▪^,^40^^▪▪^]. Alternatively, close serum monitoring with dose titration as needed may be employed [9^▪▪^,^10^^▪▪^].

Maternal serum lithium should be assessed within 24–48 h postpartum and thereafter at 5 to 7-day intervals (or biweekly) during the first month [9^▪▪^,^10^^▪▪^,^39^^▪▪^,^40^^▪▪^], with a therapeutic target of 0.8–1.0 mEq/l [9^▪▪^,^10^^▪▪^]. If lithium was discontinued during pregnancy, postpartum re-initiation may be warranted. In high-risk, lithium-naïve individuals, prophylactic initiation at delivery may be considered [10^▪▪^].

Lithium has evidence for acute and maintenance efficacy in PBD; in lithium-responsive, high-risk women, continuation within a structured perinatal plan ensuring maternal and infant safety may support complex risk-prevention.

#### Breastfeeding

Some authors find breastfeeding possible with infant and drug monitoring while on lithium, if infant serum levels remain low. In breastfeeding women using lithium, at 750 mg/day mean dose, mean maternal serum levels were 0.62 mmol/l and infant levels 0.16 mmol/l [45]. Breastfed infants should be observed for changes in general condition, irritability, somnolence, altered muscle tone, feeding difficulties, or poor weight gain, considering regular assessment of serum lithium, cardiac, thyroid, and renal function, and prompt evaluation if concerns arise [9^▪▪^,^39^^▪▪^,^40^^▪▪^].

French expert group advises against lithium use during lactation, discourages Hale's L4–L5 agents, and recommends L2 agents (e.g., lamotrigine, quetiapine, and olanzapine) as acceptable alternatives [35^▪▪^]. For preterm, medically fragile, or ill newborns of mothers taking lithium, breastfeeding is consistently discouraged [40^▪▪^].

Breastfeeding during lithium therapy remains controversary, requiring close collaboration with healthcare providers, maternal vigilance for potential adverse effects, pediatric follow-up, and early recognition of complications with prompt access to care services.

### Antipsychotics

#### Pregnancy and peripartum

The role of antipsychotics in perinatal relapse prevention requires further study, given pharmacokinetic changes and high postpartum relapse risk. Compared with lithium, they may offer lower toxicity, simpler monitoring, and beneficial sedation [10^▪▪^]. Prenatal exposure is associated with maternal weight gain, metabolic effects, gestational diabetes, and fetal growth differences, but not with major malformations, preterm birth, or long-term neurodevelopmental harm; transient neonatal adaptation syndrome and early motor delays can occur [10^▪▪^,^40^^▪▪^].

#### Breastfeeding

Olanzapine and quetiapine are first-line agents during lactation [35^▪▪^,^39^^▪▪^,^46^]. Risperidone and aripiprazole show low milk transfer; however, risperidone has more reported neonatal adverse effects, and aripiprazole carries a risk of impaired lactation [40^▪▪^]. Second-generation agents – excluding clozapine – are generally considered compatible with breastfeeding in full-term, healthy infants, with monitoring for sedation, feeding difficulties, abnormal weight gain, extrapyramidal symptoms, and developmental delay [46]. Dopamine partial agonists may impair lactogenesis, although neonatal and maternal factors (e.g., poor latching, prematurity, health complications) may contribute, findings align with prolactin-lowering effect [47^▪^,^48^].

## LONG-ACTING INJECTABLE ANTIPSYCHOTICS

Safety data are very limited about the use of LAI in the perinatal period. Continuation might be considered in women with poor adherence or frequent relapses, with close fetal and neonatal monitoring [10^▪▪^,^49^].

## ANTIDEPRESSANTS AND BENZODIAZEPINES

In a cohort study of pregnant woman with bipolar disorder, antidepressants were used in 27%, nearly half without a mood stabilizer [50], though antidepressant monotherapy is discouraged in bipolar disorder due to the heightened risk of mania [14,40^▪▪^]. During lactation, most antidepressants result in clinically insignificant infant exposure and rare, typically mild adverse effects [51,52].

Perinatal use of benzodiazepines and Z-hypnotics appear well tolerated in appropriate doses and short-term use, with low infant exposure and rare, mild adverse effects, though fetal and neonatal monitoring remains essential [10^▪▪^,^40^^▪▪^,^51^].

## NEW AGENTS

Novel agents (lumateperone, OLZ/SAM, ketamine/esketamine), and experimental anti-inflammatory or NMDA-targeting drugs show promise for specific bipolar disorder subgroups [53]. Acute PPP and early-onset severe PPD are related to BPS through shared inflammatory and immune dysregulation, suggesting future potential for anti-inflammatory and T-cell enhancing strategies [54].

## ELECTROCONVULSIVE THERAPY (ECT)

ECT is a rapid and effective treatment for severe or treatment-resistant PMD in high-risk cases – suicide/infanticide, catatonia – or poor medication response, and generally compatible with breastfeeding [55,56]. Side effects are mostly mild and transient (cognitive disturbances, memory problems, confusion), while serious complications are rare. Although national data show postpartum ECT use is infrequent, it yields high remission rates, while relapse and readmission remain common, underscoring the need for maintenance treatment and follow-up [33,57].

## PSYCHOSOCIAL INTERVENTIONS

Integrating patients lived experience in care and involving peer experts, represents a growing trend.

A review co-produced with experts by experience highlights PPD is marked by sudden emotional collapse, loneliness, guilt, and identity loss, while PPP involved perceptual disturbances, disorganized thinking, and at times self-harm or infant-harm thoughts. Stigma, sociocultural pressures, and lack of awareness worsen isolation and delay care [58^▪▪^].

In a study of mothers with bipolar disorder, positive perceptions of motherhood were linked to euthymia/mild symptoms and maternal achievement, while mixed perceptions reflected higher symptom burden and struggles with motherhood. Challenges included balancing self- and infant care, family strain, and limited healthcare support, whereas illness acceptance, mindfulness, and partner/family support were protective. Postpartum care should both addresses mood stabilization and demands of early motherhood [59]. Integrated psychosocial and pharmacological care remains essential to prevent postpartum relapse, while there is an evidence gap for nonpharmacological methods in PBD [60].

Enhancing social support and reducing parental stress is crucial. Evidence-based methods (CBT, IPT, mindfulness-based CBT, IPSRT) show efficacy in stabilizing interpersonal functioning and daily routines, including sleep patterns [9^▪▪^].

Psychosocial support in PPP builds on evidence from nonperinatal psychosis. Key elements include psychoeducation, CBT, interpersonal and family-focused therapy, and peer/support groups. Preconception counselling and education aid early intervention, while postacute care should prioritize mother-infant bonding and parent-infant psychotherapy [33].

## CONCLUSION

Bipolar disorder carries a high risk of perinatal relapse/onset, especially in the first weeks postpartum. Early detection and management are crucial for optimal outcome, within a life-course approach. Lithium remains the most effective treatment. Well tolerated use requires maternal and infant monitoring. Lamotrigine and atypical antipsychotics offer safer, though potentially less effective alternatives. Drug-exposed infants need close monitoring for fetal, neonatal, and developmental concerns. Optimal care combines pharmacological and psychosocial strategies, sleep protection, early assessment of mother-infant interactions, family focused view, and access to mother-baby units/integrated care. Future research should integrate lived experience, refine nonpharmacological approaches, clarify child outcomes, and evaluate emerging therapies.

## Acknowledgements

*The authors would like to thank Mátyás Péter Eötvös, the librarian, North-Buda Saint John Central Hospital, and Ildikó Danis, PhD, Senior Research Fellow, Semmelweis University, Institute of Mental Health for their technical assistance in the literature research.*

https://www.dropbox.com/scl/fi/kfjggz6h2j5gwml3zkfyf/Video-abstract_State-of-the-Art-treament_PBD_TKurimay-et-al.mp4?rlkey=t29g4y7io219qdz5xc3fh50gf&st=o7l4g3q8&dl=0

### Financial support and sponsorship

*None.*

### Conflicts of interest

*There are no conflicts of interest.*

## REFERENCES AND RECOMMENDED READING

Papers of particular interest, published within the annual period of review, have been highlighted as:

▪ of special interest
▪▪ of outstanding interest
